# ﻿New and little-known stonefly species of the *Rhopalopsolevietnamica* ‘western assemblage’ group (Plecoptera, Leuctridae) from China

**DOI:** 10.3897/zookeys.1183.103288

**Published:** 2023-11-06

**Authors:** Mengyu Li, Bingli Wang, Ding Yang, Dávid Murányi, Weihai Li, Hongliang Wang

**Affiliations:** 1 Henan International Joint Laboratory of Taxonomy and Systematic Evolution of Insecta, Henan Institute of Science and Technology, Xinxiang, Henan 453003, China Henan International Joint Laboratory of Taxonomy and Systematic Evolution of Insecta, Henan Institute of Science and Technology Xinxiang China; 2 Department of Entomology, China Agricultural University, 2 Yuanmingyuan West Road, Beijing 100193, China China Agricultural University Beijing China; 3 Department of Zoology, Eszterházy Károly Catholic University, Leányka u. 6, Eger H-3300, Hungary Eszterházy Károly Catholic University Eger Hungary

**Keywords:** female description, new species, redescription, *Rhopalopsolebawanglinga*, *Rhopalopsoledentiloba*, *Rhopalopsolehainana*, taxonomy

## Abstract

We examined Chinese stonefly specimens of the *Rhopalopsolevietnamica* ‘western assemblage’ group. A new species from Hainan Province, *R.bawanglinga* Li, Li & Yang, **sp. nov.** is described and illustrated from male and female adults, and it is compared to closely related taxa. The hitherto unknown female of *R.hainana* Li & Yang, 2010 is described. Morphological evidence is presented for the identity of *R.dentiloba* Wu, 1973, on the basis of topotypes from Yunnan Province, southwestern China.

## ﻿Introduction

The stonefly genus *Rhopalopsole* Klapálek, 1912 is one of the largest leuctrid genera, with about one hundred valid species, distributed in the Oriental and East Palaearctic regions (DeWalt et al. 2023). Over sixty species have been recorded from China by Chen et al. (2022), Chen and Du (2017), Klapálek (1912), Li et al. (2010a, 2010b, 2011, 2017, 2022), Li and Yang (2010, 2011, 2012), Mo et al. (2018), Qian et al. (2014), Qian and Du (2011, 2012a, 2012b, 2013, 2017), Sivec et al. (2008), Wu (1949, 1973), Yang and Du (2021a, 2021b, 2022), Yang et al. (2021, 2022), Yang et al. (2004, 2009), Yang and Li (2006) and Yang and Yang (1991a, 1991b, 1993, 1994, 1995a, 1995b). The ‘western assemblage’ of the *R.vietnamica* group was recognized by Sivec et al. (2008), and currently, thirteen species belong to this group (Wu 1973; Yang and Yang 1993; Sivec et al. 2008; Li and Yang 2010; Li et al. 2017, 2010b, 2022; Qian and Du 2017; Yang et al. 2022; Yang and Du 2022). In this contribution, two species of this species group were described or supplemented, including a new species, *R.bawanglinga* Li, Li & Yang, sp. nov. from Hainan Province in southern China, and the hitherto unknown female of *R.hainana* Li & Yang, 2010. Additionally, we provide a redescription and new images for *R.dentiloba* Wu, 1973, a newly included species of this group, based on topotypes.

**Table 1.** World list of species belonging to the *Rhopalopsolevietnamica* ‘western assemblage’ group. Abbreviation: M, male; F, female.

Species	Known life stages	Distribution
*R.amamiensis* Kawai, 1967	M, F	Japan: Kagoshima Prefecture, Okinawa
*R.assamensis* Sivec & Harper, 2008	M, F	India: Assam
*R.bawanglinga* sp. nov.	M, F	China: Hainan
*R.brevidigitata* Qian & Du, 2017	M	China: Yunnan
*R.dentiloba* (Wu, 1973)	M, F	China: Yunnan
*R.fengyangshanensis* Yang, Shi & Li, 2009	M	China: Fujian, Zhejiang
*R.furcospina* (Wu, 1973)	M, F	China: Guangxi, Shaanxi, Sichuan, Zhejiang
*R.hainana* Li & Yang, 2010	M	China: Hainan
*R.nanlinga* Yang & Du, 2022	M	China: Guangdong
*R.sinensis* Yang & Yang, 1993	M	China: Chongqing, Fujian, Gansu, Guangxi, Guizhou, Hubei, Ningxia, Shaanxi, Zhejinag; Vietnam: Laocai
*R.singiplatta* Yang & Du, 2022	M, F	China: Sichuan
*R.sipirokana* Sivec & Harper, 2008	M, F	Sipirok: Sumatra
*R.vietnamica* Sivec & Harper, 2008	M, F	Vietnam: Thinhoa
*R.yajunae* Li & Yang, 2010	M	China: Zhejiang
*R.yunnana* Sivec & Harper, 2008	M, F	China: Yunnan

**Table 2.** China, Hainan Province specimens used for COI based identification, genetic differentiation, and circumscription analyses of three *Rhopalopsole* species.

Species	Sex	Catalog number	GenBank accessions	Geographic coordinates
*R.hainana*	1♀	J61	OR435219	19°4'52.32"N, 109°31'10.92"E
*R.hainana*	1♀	J64	OR435220	19°4'52.32"N, 109°31'10.92"E
*R.hainana*	1♂	J66	OR435221	19°4'52.32"N, 109°31'10.92"E

## ﻿Material and methods

The specimens were collected by hand and preserved in 75% ethanol. Most of the studied materials are deposited in the Entomological Museum of China Agricultural University, Beijing, China (**CAU**). Additional specimens are deposited in the Insect Collection of Henan Institute of Science and Technology, Xinxiang, Henan Province, China (**HIST**), as indicated in the text. The color illustrations were made using an Imaging Source CCD attached to a Leica M205FA dissecting microscope. The morphological terminology follows that of Sivec et al. (2008). The association of sexes of *Rhopalopsolehainana* is based on the type locality and barcoding data.

The maps were downloaded from Standard Map Service (http://bzdt.ch.mnr.gov.cn/) and edited by Photoshop 2020, map number is GS (2019)1823.

Total genomic DNA was extracted from thorax muscle using the TIANamp Genomic DNA kit (Tiangen Biotechnology, Beijing, China) following the manufacturer’s protocol. The universal primers LCO1490 and HCO2198 (Folmer et al. 1994) were used to amplify the target COI gene region. Polymerase chain reaction (PCR) program was as follows: initial denaturation at 95 °C for 60 s, followed by 40 cycles of 30 s at 95 °C, 50 s at 40–60 °C, and 60 s at 65 °C, and a final extension phase of 65 °C for 10 min. The PCR products were confirmed by 1% agarose gel electrophoresis and then sent to Sangon Biotechnology Co. Ltd. (Shanghai, China) for DNA sequencing. The primary sequences were assembled using Contigexpress software (Vector NTI Informax). Genetic distances between sequences were calculated in MEGA v.5.2 and bootstrap analysis was conducted using 1000 replicates. The GenBank accession numbers for the three specimens are in Table 2.

## ﻿Results and discussion

**Family Nemouridae Billberg, 1820**

**Genus *Rhopalopsol* e Klapálek, 1912**

### ﻿ Rhopalopsole bawanglinga

Taxon classificationAnimaliaPlecopteraLeuctridae

Li, Li & Yang sp. nov.

330448BE-F47F-5838-820F-8FA6835AA1D3

https://zoobank.org/F26FAD81-D6AA-44BA-99B7-88067CD05AB1

Figs 1 , 2

#### Type material.

***Holotype***: male (CAU), China: Hainan Province, Changjiang County, Bawangling National Forest Park, Dong’er Station, 22.X.2007, 19°15'1.44"N, 109°2'2.4"E, 1000 m, leg. D. Yang. ***Paratypes***: 1 female (CAU): same data as holotype; 2 females (HIST), China: Hainan Province, Ledong County, Jianfengling, Tianchi–Mingfenggu, 3.VIII.2016, 18°44'49.2"N, 108°50'57.12"E, 890 m, leg. Weihai Li, Rongfeng Wang.

#### Diagnosis.

Male adult of this species is characterized by the lateral projections of the tergum 10 being bifurcate with the upper spine longer than the lower one in lateral view. The cercus bears a stout dorsoapical spine, and the epiproct is subtrapezoid with deep, circularly incised anterior margin in dorsal view. Females are diagnosed by the posterior margin of sternum 7 forming a narrowly produced pregenital plate, posteromedial portion slightly bilobed.

#### Description.

***Adult habitus*.** Forewing length is 6.8 mm in the male, 7.0–7.4 mm in the females. Head brown to dark brown, slightly wider than pronotum; compound eyes black; antennae and mouthparts brownish to brown. Pronotum brown with dark rugosities; wings subhyaline with darker veins; legs brown. Abdomen brown.

**Figure 1.**
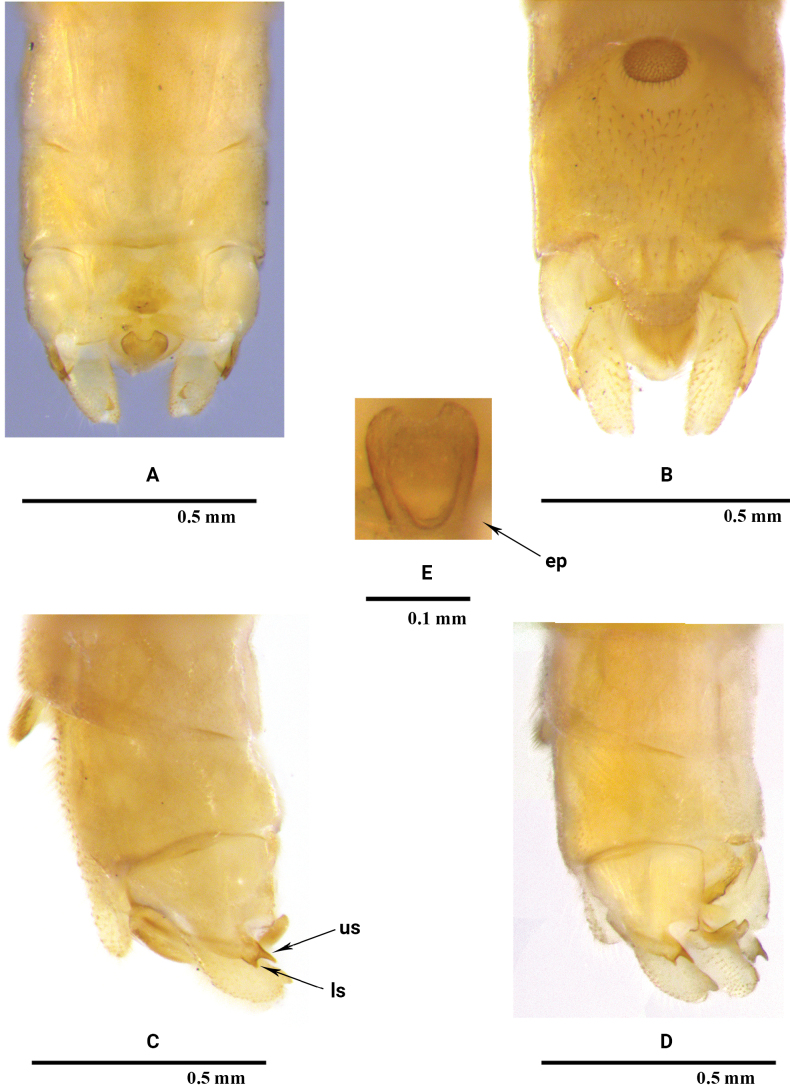
*Rhopalopsolebawanglinga* Li, Li & Yang, sp. nov. (male) **A** terminalia, dorsal view **B** terminalia, ventral view **C** terminalia, lateral view **D** terminalia, dorsolateral view **E** epiproct dorsal view. **ep**: epiproct; **ls**: lower spine; **us**: upper spine.

**Male** (Figs 1, 3). Tergum 9 (Figs 1A, 3A) weakly sclerotized except anterior margin and lateral portions, medial 1/3 with a large trapezoidal membranous area surrounded by lateral sclerotized portion and ornamented posteromedial margin which terminates in a thin, produced sclerite with tiny granules. Sternum 9 (Figs 1B, 3B) longer than wide, distal portion with distinct trapezoidal subgenital plate, the plate about as long as wide, with a subapical constriction; vesicle dark brown, ovum-shaped in ventral view, tongue-like in lateral view, covered with dense hairs. Tergum 10 (Figs 1A, C, 3A, C) with well sclerotized, bifurcate lateral projections, terminating in two acute, parallel-sided points in dorsal aspect, the upper spine is longer than lower spine in lateral view. Central plate (Figs 1A, D, 3A, D) sclerotized and trilobed, lateral lobes subtriangular, together with a circular anteromedial incision of the medial lobe resemble flying wings; medial lobe darkly pigmented, with a small knob-like projection. Transverse bars slightly elevated medially, triangular, posterior margin and inner portion sclerotized. Cercus barely 3× longer than wide, curved dorsally, with a stout subapical spine. Epiproct (Figs 1A, E, 3A, E) strongly sclerotized and darkly sclerotized laterally, subtrapezoid with deep, circularly incised anterior margin in dorsal view, longer than wide. Subanal lobe (Figs 1B, 3B) distinctly sclerotized basally, lateral margins and apex membranous, ventral furrows vague.

**Figure 2.**
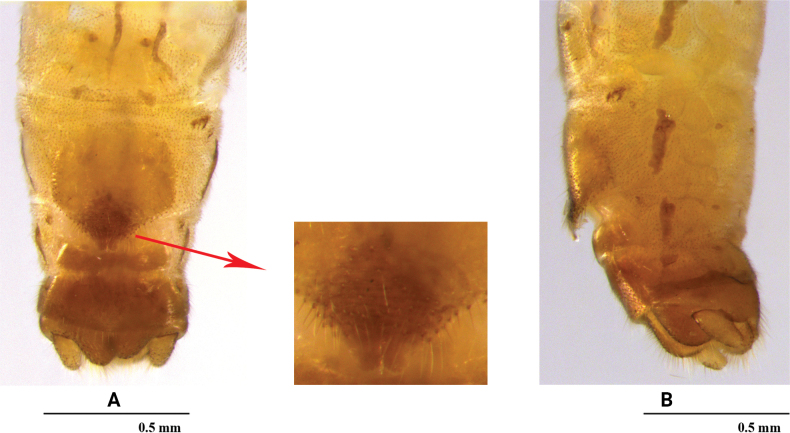
*Rhopalopsolebawanglinga* Li, Li & Yang, sp. n. (female) **A** terminalia, ventral view (apex of pregenital plate enlarged) **B** terminalia, lateral view.

**Female** (Fig. 2A, B). Posterior margin of sternum 7 forming a triangularly produced pregenital plate; plate with sclerotized bilobed distal tip. Sternum 8 membranous except a slender, transverse posterior strip.

**Figure 3.**
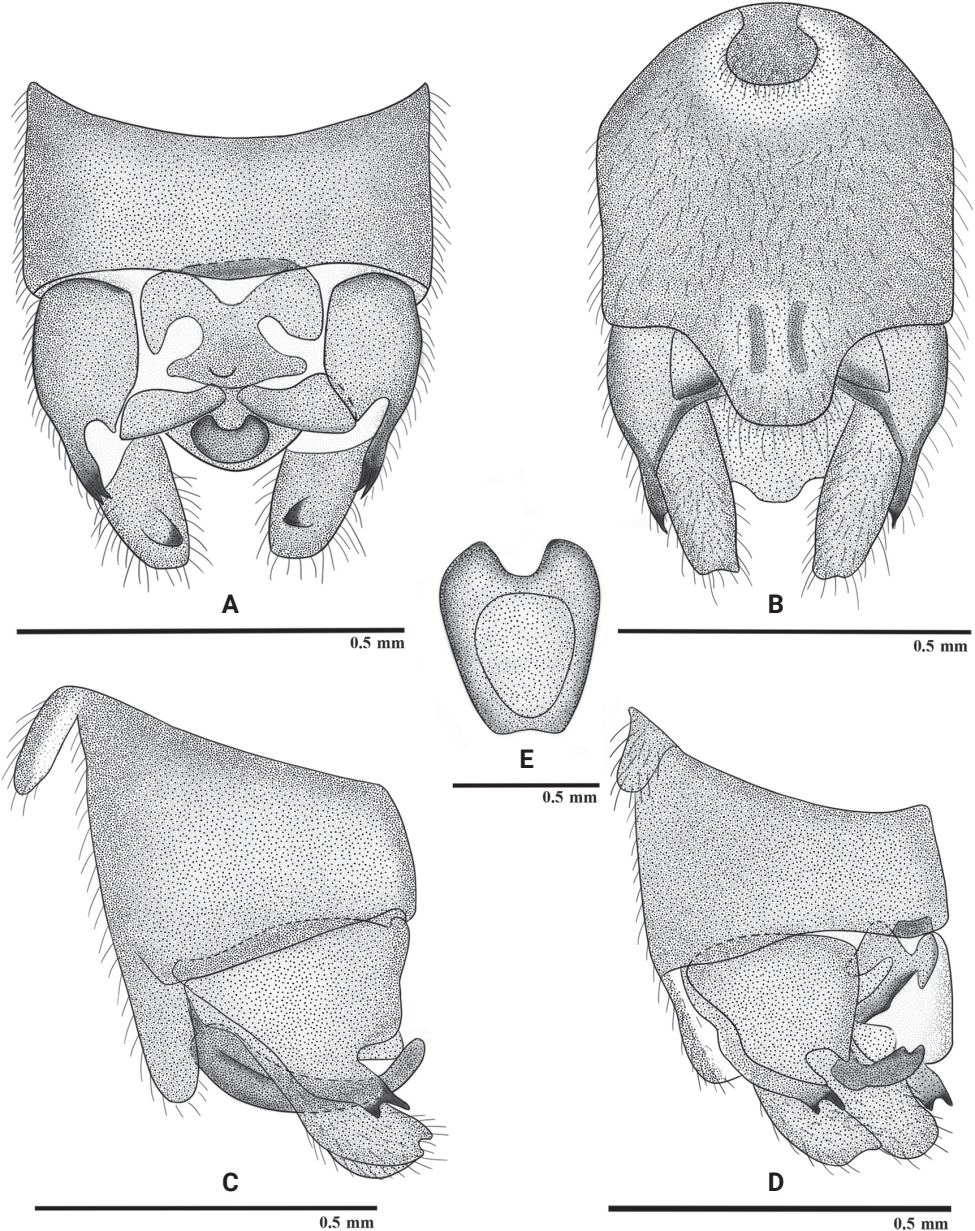
*Rhopalopsolebawanglinga* Li, Li & Yang, sp. n. (male) **A** terminalia, dorsal view **B** terminalia, ventral view **C** terminalia, lateral view **D** terminalia, dorsolateral view **E** epiproct dorsal view. **ep**: epiproct; **ls**: lower spine; **us**: upper spine.

#### Etymology.

The specific name refers to the Bawangling National Forest Park.

#### Distribution.

China (Hainan).

#### Remarks.

The new species is a typical member of the *R.vietnamica* group, western assemblage sensu Sivec et al. (2008). The male of the new species seems closely related to *R.hainana* from Hainan and *R.cestroidea* Li & Yang, 2017 (in: Li et al. 2017) from Guangxi, as they share a similar wide epiproct. However, it can be differentiated from both by bifurcate lateral projections on tergum 10, with the upper spine being longer than the lower one in lateral view, and the presence of a concave anterior margin of the epiproct. In *R.hainana* and *R.cestroidea*, the terminal bifurcation of lateral projections on tergum 10 is of equal length in lateral view, and the epiproct has a truncate or convex anterior margin. Additionally, the cercus of *R.cestroidea* lacks a spine, and that of *R.hainana* has a sharp subapical spine, whereas the cercal spine in the new species is stout. The female is distinctive due to the bilobed distal tip of the pregenital plate.

### ﻿ Rhopalopsole hainana

Taxon classificationAnimaliaPlecopteraLeuctridae

Li & Yang, 2010

471B654B-1D16-5DB4-8F55-A5442CBAE2CC

Fig. 4

Rhopalopsole hainana Li & Yang, 2010: 59 (original description).

#### Material examined.

11 males, 5 females (CAU), China: Hainan Province, Baisha, Yinggeling, Hongxin Village, 23–24.V.2007, 19°12'22.32"N, 109°33'14.4"E, 250 m, leg. J.X. Liu; 1 female (CAU), China: Hainan Province, Lingshui County, Diaoluo Mountain, 1.VII.2011, 18°43'31.08"N, 109°52'8.4"E, 294 m, leg. W.H. Li; 2 females (CAU), China: Hainan Province, Baisha, Yinggeling, 29.X.2011, 19°4'52.32"N, 109°31'10.92"E, 1191 m, leg. W.H. Li.

#### Description.

**Figure 4.**
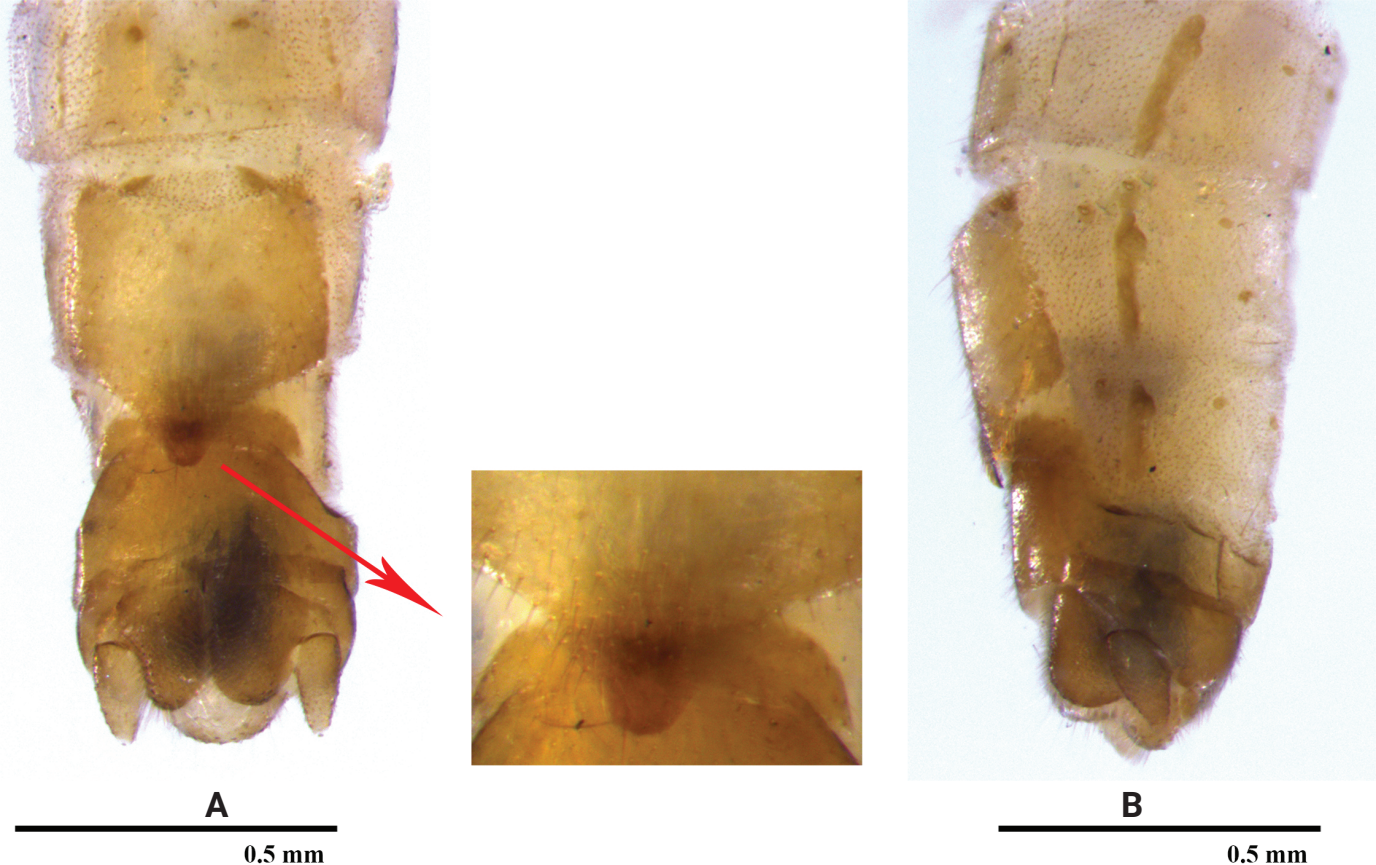
*Rhopalopsolehainana* Li & Yang, 2010 (female) **A** terminalia, ventral view (apex of pregenital plate enlarged) **B** terminalia, lateral view.

**Female.** (Fig. 4A, B) Pregenital plate on sternum 7 sclerotized, distally produced into strongly sclerotized triangular lobe with sinuous lateral margins, covered with long hairs; dorsoventrally flattened in lateral view. Sternum 8 with a trapezoid, transverse band across the posterior half of the segment.

#### Remarks.

Genetic distance refers to the genetic divergence between species and can be used to compare the genetic similarity between difference species (Wang et al. 2013); generally, more than 98% of congeneric species or sister species pairs have greater than 0.02 sequence divergence (Johns and Avise 1998; Hebert et al. 2003; Park et al. 2011). In our study, pairwise distances of the *Rhopalopsole* species were 0–0.9%; lower than the 2% threshold considered for a rough differentiation between intraspecific and interspecific distances (Zhou et al. 2009). The genetic distance of COI between J66 and J61 is 0 and between J66 and J64 is 0.9% (Table 3). The combination of morphology and molecular data suggest the female is *R.hainana*.

**Table 3.** Genetic distances among sequenced specimens. Upper (right) triangular matrix: standard deviation; lower (left) triangular matrix: genetic distances.

	J61 F *R.hainana*	J64 F *R.hainana*	J66 M *R.hainana*
J61 F *R.hainana*		0.006	0.0
J64 F *R.hainana*	0.009		0.006
J66 M *R.hainana*	0.0	0.009	

We compared the holotype with the specimens from Baisha County, which originated from near the type locality. The hitherto unknown female resembles the female of *R.bawanglinga*, but the tip of the pregenital plate is not bilobed, and the transverse band on sternum 8 is more pronounced.

### ﻿ Rhopalopsole dentiloba

Taxon classificationAnimaliaPlecopteraLeuctridae

Wu, 1973

9AF174B0-7237-58A6-BB64-2952881AC864

Figs 5 , 6

Rhopalopsole dentiloba Wu, 1973: 105 (original description).

#### Material examined.

4 males, 1 female (CAU), China: Yunnan Province, Xishuangbanna, Meng shimron botanical garden, 22.IV.2007, 21°55'9.12"N, 101°16'6.96"E, 550 m, leg. D. Hui. 1 male (HIST), China: Yunnan Province, Xishuangbanna, Mengla, Wangtianshu scenic spot, 9.V.2009, 22°1'53.76"N, 100°52'32.88"E, 840 m, leg. X.S. Yang.

#### Remarks.

**Figure 5.**
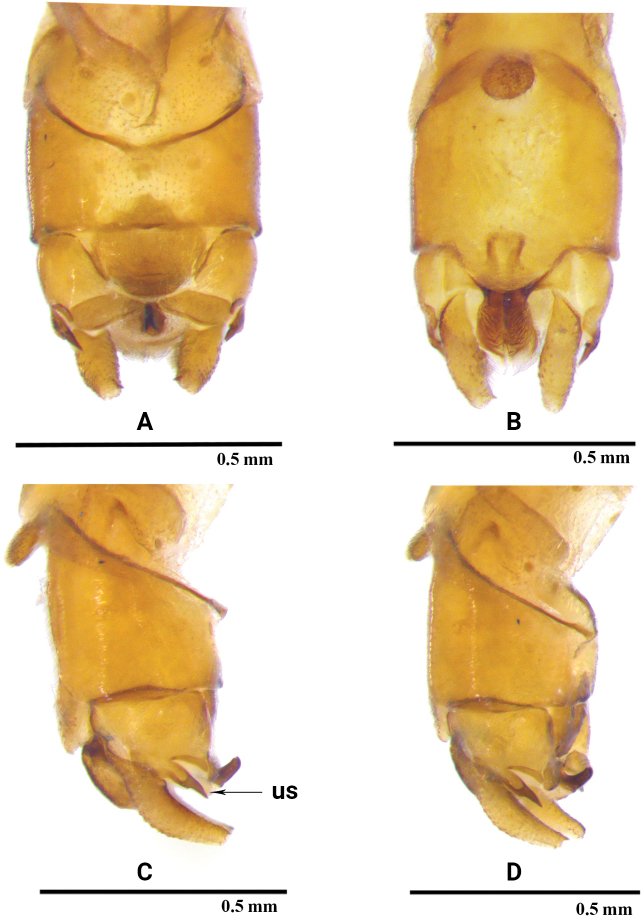
*Rhopalopsoledentiloba* Wu, 1973 (male) **A** terminalia, dorsal view **B** terminalia, ventral view **C** terminalia, lateral view **D** terminalia, dorsolateral view. **us**: upper spine.

**Figure 6.**
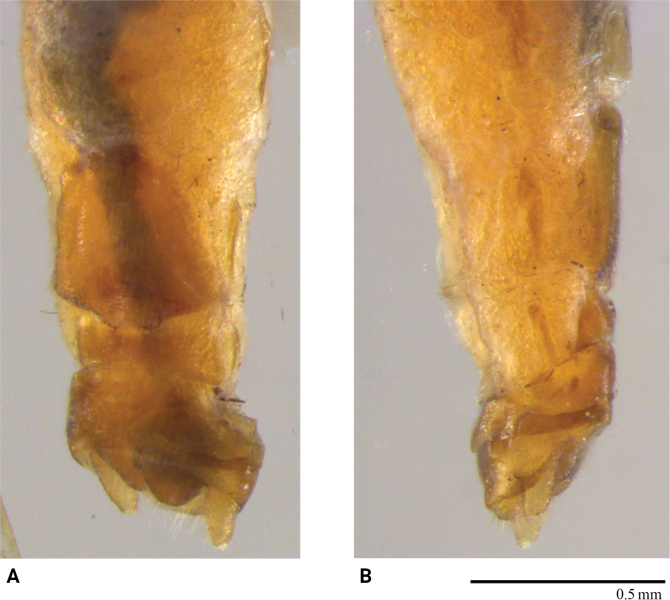
*Rhopalopsoledentiloba* Wu, 1973 (female) **A** terminalia, ventral view **B** terminalia, lateral view.

We examined several specimens from Xishuangbanna, Yunnan Province, which is the same location where *R.dentiloba* was described. The original description and illustrations did not provide sufficient details for identification. Therefore, we discuss herein the distinctive morphological characters of our specimens. This species is a typical member of the *R.vietnamica* group, western assemblage sensu Sivec et al. (2008). The male tergum 9 (Fig. 5A, C, D) has a small triangular, upraised sclerotized process on hind margin. Tergum 10 (Fig. 5A, B) bears a small bifurcate spine at each posterolateral corner in dorsal and lateral aspects, but the inner/lower spine is not easily observed. In lateral view (Fig. 5C, D), unbranched, sclerotized lateral projections are seen. The epiproct is small, hook-like. The minute teeth of the subanal lobe described for the holotype are possibly the distinct furrows seen in the males observed in this study. The identity of these specimens is primarily based on a similar female and their proximity to the type locality (Mengsong town or Mengsong village in this town). The female (Fig. 6A, B) is identical to the original illustration. However, doubts may arise about association of the original female and the male holotype, as tropical Yunnan is well known for its high biodiversity, where more *Rhopalopsole* species may co-occur.

## ﻿Conclusion

**Figure 7.**
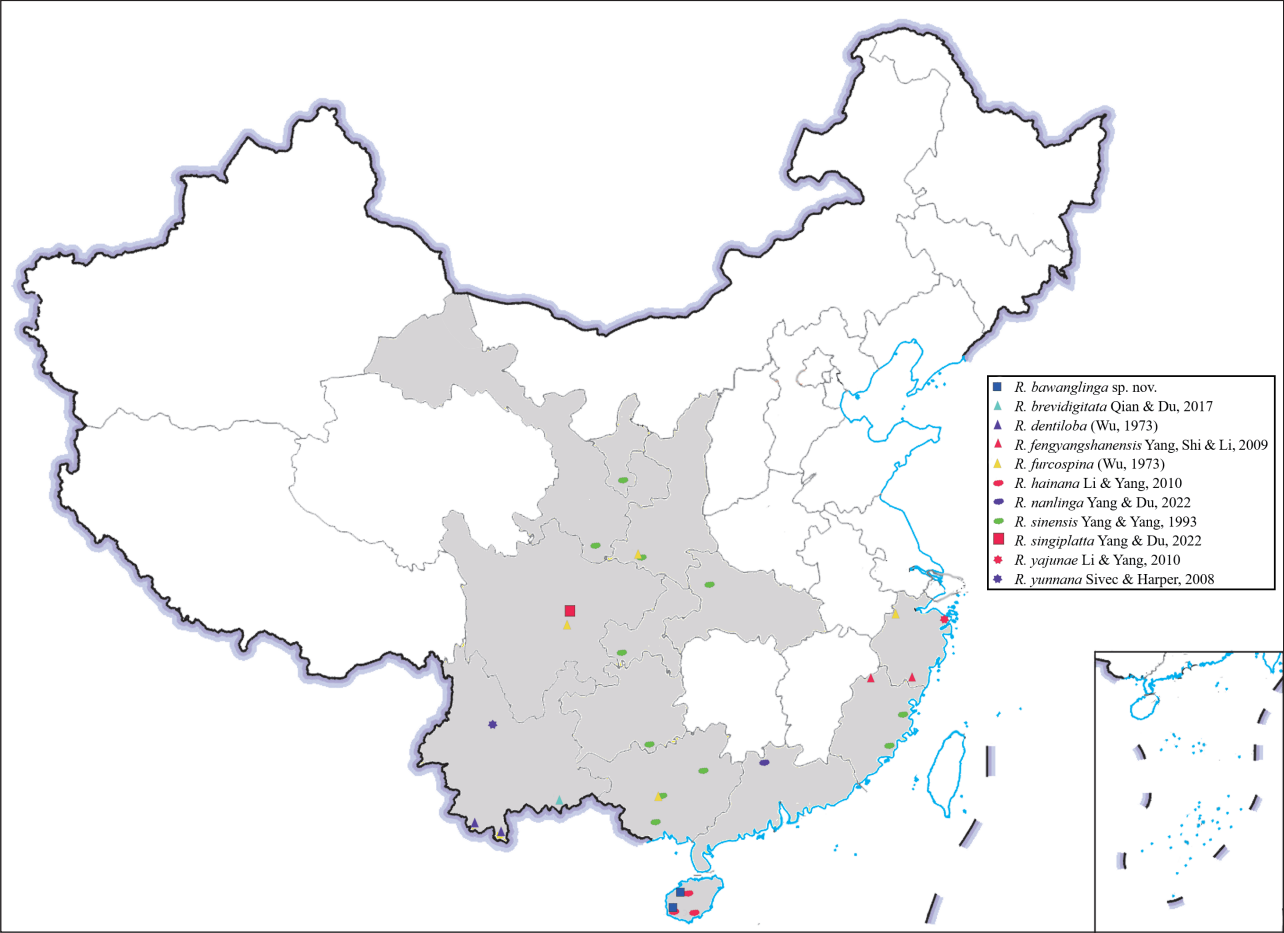
Distribution of species of the *Rhopalopsolevietnamica* ‘western assemblage’ group of China. City records shaded in grey.

Thirteen species were previously recorded in the *R.vietnamica* ‘western assemblage’ group, as specified in Table 1. *Rhopalopsoledentiloba* was included in the *R.shaanxiensis* species group but is now transferred to the *R.vietnamica* ‘western assemblage’ group due to its great similarities to *R.sinensis* Yang & Yang, 1993. Considering the geographical (Fig. 7) differences between populations of *R.sinensis*, molecular methods should be applied to confirm the status of *R.dentiloba* in the future. With the description of the new taxon and the inclusion of *R.dentiloba*, the number of species in this group in China is now up to eleven, and more new species may await discovery.

## Supplementary Material

XML Treatment for Rhopalopsole bawanglinga

XML Treatment for Rhopalopsole hainana

XML Treatment for Rhopalopsole dentiloba

## ﻿Additional information

### Author ORCIDs

Mengyu Li https://orcid.org/0000-0002-7950-4142

Bingli Wang https://orcid.org/0000-0002-1080-549X

Ding Yang https://orcid.org/0000-0002-7685-3478

Dávid Murányi https://orcid.org/0000-0002-3907-5590

Hongliang Wang https://orcid.org/0000-0002-9758-1328

### Data availability

All of the data that support the findings of this study are available in the main text.
